# Comparison of the Probabilistic Ant Colony Optimization Algorithm and Some Iteration Method in Application for Solving the Inverse Problem on Model With the Caputo Type Fractional Derivative

**DOI:** 10.3390/e22050555

**Published:** 2020-05-15

**Authors:** Rafał Brociek, Agata Chmielowska, Damian Słota

**Affiliations:** Department of Mathematics Applications and Methods for Artificial Intelligence, Silesian University of Technology, Kaszubska 23, 44-100 Gliwice, Poland; agata.chmielowska@polsl.pl (A.C.); damian.slota@polsl.pl (D.S.)

**Keywords:** fractional derivative, fractional differential equation, inverse problem, mathematical modeling, heat conduction

## Abstract

This paper presents the algorithms for solving the inverse problems on models with the fractional derivative. The presented algorithm is based on the Real Ant Colony Optimization algorithm. In this paper, the examples of the algorithm application for the inverse heat conduction problem on the model with the fractional derivative of the Caputo type is also presented. Based on those examples, the authors are comparing the proposed algorithm with the iteration method presented in the paper: Zhang, Z. An undetermined coefficient problem for a fractional diffusion equation. *Inverse Probl.* 2016, *32*.

## 1. Introduction

The inverse problems are an important and commonly encountered class of problems in many branches of technology and mathematics [1,2]. Solving them involves in the appropriate choice of the input parameters of the model (e.g., material coefficients or initial-boundary conditions) in order to model the process in such a way that the suitable output is obtained.

In this paper, we consider the model with the fractional differential equation with the fractional derivative [3,4] of the Caputo type. This type of derivative is being used to describe the phenomenon of the anomalous diffusion. The example of this kind of phenomena is a heat conduction in the porous material. Voller [5] shows that for the materials of anomalous porosity, the models containing the fractional derivative are better for describing the problem that the models with the classical derivative. The experiments conducted in the papers [6,7] show that the fractional derivatives make sense when describing the phenomenon of the heat conduction in such materials as the composites or the porous aluminum. In [6], the authors consider the inverse heat conduction problem in the composite material. For the model presented in this paper the fractional derivative of the Caputo type was used. In [7], the authors are comparing the mathematical models containing various derivatives (classical, fractional of the Riemann–Liouville type and fractional of the Caputo type) basing on the experimental data obtained for the porous aluminum. Moreover, in this case the fractional derivative turned out to be better for describing the phenomenon. More on the application of the fractional calculus in various fields of science can be found in the papers [8,9,10,11,12,13,14].

In this paper, we present the algorithm for solving the inverse problem on the model containing the fractional derivative of the Caputo type. In the presented model, the thermal conductivity coefficient is being reconstructed. For solving the direct problem the finite difference method with the approximation of the Caputo type derivative [15] was used. The algorithm for solving the inverse problem consists in minimizing the functional representing the approximation error. For this purpose, one of the algorithms of the swarm intelligence was used [16,17]. In this paper, the numerical examples used for comparison of the presented algorithm with the algorithm shown in [18] are also being presented.

## 2. Formulation of the Problem

We consider two methods for solving the inverse problem on the models containing the fractional derivatives. Let us consider the following differential equation with the fractional derivative and the initial-boundary conditions.

(1) $$c\varrho \frac{\partial^{\alpha }u\left(x,t\right)}{\partial t^{\alpha }}=\lambda \left(t\right)\frac{\partial^{2}u\left(x,t\right)}{\partial x^{2}}+g\left(x,t\right),$$

(2) u(x,0)=f(x),x∈[0,L],

(3) u(0,t)=ϕ(t),t∈(0,T],

(4) u(L,t)=ψ(t),t∈(0,T].

In the above Equation there is a fractional derivative with respect to time. In this paper, this derivative is of order α∈(0,1) and of a Caputo type which is defined as follows,

(5) $$\frac{\partial^{\alpha }u\left(x,t\right)}{\partial t^{\alpha }}=\frac{1}{\Gamma (1-\alpha )}\int_{0}^{t}\frac{\partial u(x,s)}{\partial s}{\left(t-s\right)}^{-\alpha }\;ds.$$

Equation (1) (with the fractional derivative of order α with respect to time) with the initial-boundary conditions (2)–(4) may be used to model the heat conduction phenomenon in the porous material. Then, the respective components of the model take the following names.

x,t—spatial and time variable,
*u*—function representing the temperature distribution,
*c*—specific heat,
ρ—density,
λ—thermal conductivity,
α—order of fractional derivative,
*g*—additional heat source,
*f*—function representing the initial condition,
ϕ,ψ—functions representing the boundary condition of the first type.

Generally speaking, the solution of the inverse heat conduction problem consists in such a choice of the model parameters (model input) so the given output temperature distribution is obtained. More information regarding the heat processes modeling in the porous materials, inverse heat conduction problems, and use of the fractional calculus for modeling these kind of processes may be found in [5,7,19,20,21].

### Description of the Considered Inverse Problem

In this paper, we consider two algorithms for solving the inverse problem. The description of the first method cas be found in the paper [18]. This method is iterative so in this paper we will call it the iteration method. The second algorithm is the algorithm proposed by the authors of this paper and it is based on the Real Ant Colony Optimization algorithm [16], which is the swarm artificial intelligence algorithm. The details of the algorithm are being described in the Section 3.2. More about application of the swarm intelligence algorithms can be found in [17,22,23].

In the example the thermal conductivity dependent on time λ coefficient which occurs in the Equation (1) is being reconstructed. The additional information on the basis of which the coefficient λ is being searched is the condition

$$-\lambda \left(t\right)\left(\frac{\partial u}{\partial x}\left(0,t\right)-a\left(t\right)\right)=b\left(t\right),\;\;t\in \left(0,T\right],$$

where the function *a* is given and the function *b* (the values of the function *b* at the points of the grid) is being calculated from the formula

(6) $$b\left(t^{k}\right)=b^{k}=-\lambda^{k}\left(\frac{u_{1}^{k}-u_{0}^{k}}{\Delta x}-a^{k}\right),$$

by first solving the direct problem for the exact value of the parameter λ. The values $b^{k}$ are treated as the input data for the inverse problem. For the determined thermal conductivity coefficient $\bar{\lambda }$ by solving the direct problem we obtain the values $u_{0}^{k},\;u_{1}^{k}$, and therefore we also obtain the values $\bar{b^{k}}$. By comparing the values $b^{k}$ and $\bar{b^{k}}$ we construct the algorithm:

(7) $$J\left(\bar{\lambda }\right)=\sum_{k=1}^{\bar{K}}{\left(\bar{b^{k}}-b^{k}\right)}^{2}.$$

By minimizing this functional (using the algorithm proposed by the authors) we are reconstructing the thermal conductivity coefficient λ.

## 3. Methods of Solution

In the process of solving the described inverse problem there is a need to repeatedly solve the direct problem. In this section, we first describe the solution of the direct problem and then describe the Real Ant Colony Optimization algorithm needed for minimizing the functional (7).

### 3.1. Solution of the Direct Problem

The direct problem described by the Equation (1) and the initial-boundary conditions (2)–(4) were resolved by the use of the finite difference method.

Let N,K∈N be the size of the grid respectively in the domain of space and time. We take the following grid steps; Δx=L/N,Δt=T/K. Then, the points of the grid from the space interval [0,L] are the numbers $x_{i}=i\Delta x,\;i=0,1,2,\ldots ,N$ and from the time interval [0,T] the numbers $t_{k}=k\Delta t,\;k=0,1,2,\ldots ,K$. The values of the functions f,g,u,ϕ,ψ at the grid points are denoted as follows: $f_{i}=f\left(x_{i}\right)$, $g_{i}^{k}=g\left(x_{i},t_{k}\right),\phi^{k}=\phi \left(t_{k}\right),\psi^{k}=\psi \left(t_{k}\right)$ and $u_{i}^{k}=u\left(x_{i},t_{k}\right)$. The values of the approximation function at the points $\left(x_{i},t_{k}\right)$ are denoted as $U_{i}^{k}$. The Equation (1) may be written as follows,

(8) $$\frac{\partial^{\alpha }u\left(x,t\right)}{\partial t^{\alpha }}=a\frac{\partial^{2}u\left(x,t\right)}{\partial x^{2}}+\bar{g}\left(x,t\right),$$

where $a=\frac{\lambda }{c\varrho }$ in terms of the heat conductivity is the thermal diffusivity coefficient and $\bar{g}\left(x,t\right)=\frac{g(x,t)}{c\varrho }$. The fractional derivative of the Caputo type of the order α∈(0,1) with respect to time is approximated by the following Equation [15],

(9) $$\frac{\partial^{\alpha }u\left(x,t\right)}{\partial t^{\alpha }}\approx \sigma \left(\alpha ,\Delta t\right)\sum_{j=1}^{k}\omega \left(\alpha ,j\right)\left(U_{i}^{k-j+1}-U_{i}^{k-j}\right),$$

where

$$\sigma \left(\alpha ,\Delta t\right)=\frac{1}{\Gamma \left(1-\alpha \right)\left(1-\alpha \right)\Delta t^{\alpha }},$$

$$\omega \left(\alpha ,j\right)=j^{1-\alpha }-{\left(j-1\right)}^{1-\alpha }.$$

Using the Equation (9), the boundary conditions $U_{0}^{k}=\phi^{k},\;\;U_{N}^{k}=\psi^{k},\;\;\;\;k\geq 1$ and the differential quotient for the second derivative $\frac{\partial^{2}u\left(x_{i},t_{k}\right)}{\partial x^{2}}\approx \frac{1}{{\left(\Delta x\right)}^{2}}\left(U_{i-1}^{k}-2U_{i}^{k}+U_{i+1}^{k}\right)$, after proper organizing the elements we obtain the following discrete differential equations (k≥1,i=1,2,…,N−1):

(10) $$\begin{array}{c} -\frac{a}{{\left(\Delta x\right)}^{2}}U_{i-1}^{k}+\left(\sigma \left(\alpha ,\Delta t\right)+\frac{2a}{{\left(\Delta x\right)}^{2}}\right)U_{i}^{k}-\frac{a}{{\left(\Delta x\right)}^{2}}U_{i+1}^{k}=\\ =\sigma \left(\alpha ,\Delta t\right)U_{i}^{k-1}-\sigma \left(\alpha ,\Delta t\right)\sum_{j=2}^{k}\omega \left(\alpha ,j\right)\left(U_{i}^{k-j+1}-U_{i}^{k-j}\right)+{\bar{g}}_{i}^{k}. \end{array}$$

The Equation (10) may be also written in the matrix form

(11) $$AU^{k}=D,\;\;k=1,2,\ldots ,K$$

where $U^{k}={\left[U_{1}^{k},U_{2}^{k},\ldots ,U_{N-1}^{k}\right]}^{T}$, *A* is a tridiagonal matrix of the size (N−1)×(N−1) and following coefficients, i=1,2,…,N−1,j=1,2,…,N−1,

$$a_{ij}=\left\{\begin{array}{ll} \sigma \left(\alpha ,\Delta t\right)+\frac{2a}{{\left(\Delta x\right)}^{2}}, & j=i,\\ -\frac{2a}{{\left(\Delta x\right)}^{2}} & (j=i+1\wedge i=1,2,\ldots ,N-2)\vee \\ & (j=i-1\wedge i=2,3,\ldots ,N-1), \end{array}\right.$$

and *D* is a vector ${\left[d_{1}+\frac{2a}{{\left(\Delta x\right)}^{2}}\phi^{k},d_{2},d_{3},\ldots ,d_{N-2},d_{N-1}+\frac{2a}{{\left(\Delta x\right)}^{2}}\psi^{k}\right]}^{T}$, where:

$$d_{i}=\sigma \left(\alpha ,\Delta t\right)U_{i}^{k-1}-\sigma \left(\alpha ,\Delta t\right)\sum_{j=2}^{k}\omega \left(\alpha ,j\right)\left(U_{i}^{k-j+1}-U_{i}^{k-j}\right)+{\bar{g}}_{i}^{k}.$$

### 3.2. Solution of the Inverse Problem

In order to minimize the functional (7), the Real Ant Colony Optimization algorithm [16] is being used. The inspiration for the algorithm was the swarming behavior of ants in nature. In the presented algorithm, the role of the solutions are being played by the pheromone spots in the number of *L*. At the beginning those spots are randomly placed in the considered region. Next, they are being sorted by the quality and certain probabilities are being assigned to them. The better the solution is, the higher the probability of choosing it is. This way we create the set of solutions. In every iteration each of *M* ants construct one new solution (new pheromone spot) using the probability density function (Gauss function). First the ant chooses with the certain probability the solution that will be transformed. This probability is dependent on the parameter *q* of the algorithm. If value of *q* is small then the choice of the best solution is preferred and the higher the value of *q* is, the more similar to each other the probabilities of choosing any solution are. When the solution is chosen then the ant samples its neighborhood using the Gauss function with the parameters μ and σ, where σ is dependent on the parameter ξ>0. This parameter is responsible for the disappearance of the pheromone trace. The smaller value of ξ is, the faster the algorithm converges to its best solution at the expense of further exploration of the region. Then, the set of solutions is updated with the new solutions—the new and previous solutions are being sorted by quality and the *M* of the worst solutions get rejected. Moreover, the algorithm was adapted for parallel computing. Mathematical fundaments and results concerning the convergence of the different versions of the ACO algorithm are included in [24,25,26,27]. Based on the analysis of the papers of various authors, see for example [28,29,30,31,32], as well as on the computation carried out by us (for example [7,17,20,22,31]) we come to the conclusion that using the artificial intelligence algorithms (ACO, RACO, RACO, IRM, etc.) in most cases does not require the application of the explicit regulatory procedures. There is no mathematical proof for that, only the empirical data. For the examples presented in the paper we have known the exact solutions so we could compare the approximate solutions with them. The reconstruction errors of the reconstructed parameters generally do not exceed the input data errors. They are slightly larger in some cases. It follows that the results obtained for the disturbed input data remain stable. In order to describe the algorithm we are introducing the notation

F−minimalizedfunction(theobjectivefunction),

n−numberofobjectivefunctionvariables(numberofsearchedparameters),

nT−numberofthreads,M=nT×p−numberofantsinthepopulation,

I−numberofiterations,L−numberofpheromonespots,q,ξ−algorithmparameters.

We will present the consecutive steps of the algorithm.

       **Initialization of the algorithm**

1. Setting the input parameters of the algorithm: L,M,I,nT,q,ξ.
2. Generating *L* solutions playing the role of the pheromone spots. Assigning them to the set $T_{0}$ (initial archive).
3. Computing the values of the objective function for all of the pheromone spots (parallel computing) and sorting the archive $T_{0}$ from the best solution to the worst.
         **Iteration process**
4. Assigning the probabilities to the pheromone spots according to the pattern:
  $$p_{l}=\frac{\omega_{l}}{\sum_{l=1}^{L}\omega_{l}}\;\;\;l=1,2,\ldots ,L,$$
  where weights $\omega_{l}$ are related to the solution number *l* and are expressed by the formula:
  $$\omega_{l}=\frac{1}{qL\sqrt{2\pi }}\times e^{\frac{-{\left(l-1\right)}^{2}}{2q^{2}L^{2}}}.$$
5. The ant randomly chooses the l-th solution with the probability $p_{l}$.
6. The ant transforms the *j*-th coordinate (j=1,2,…,n) of the l-th solution $s_{j}^{l}$ by sampling the neighborhood using the probability density function (in this case the Gauss function):
  $$g\left(x,\mu ,\sigma \right)=\frac{1}{\sigma \sqrt{2\pi }}\cdot e^{\frac{-{\left(x-\mu \right)}^{2}}{2\sigma^{2}}},$$
  where $\mu =s_{j}^{l},\;\sigma =\frac{\xi }{L-1}\sum_{p=1}^{L}\left|s_{j}^{p}-s_{j}^{l}\right|$.
7. Steps 5–6 are being repeated by every ant. We get *M* new solutions (pheromone spots) by that.
8. Partition of the new solutions into the nT groups. Computing the value of the objective function for the new solutions (parallel computing).
9. Adding the new solutions to the archive $T_{i}$, sorting them by the quality and rejecting the *M* worst solutions.
10. The steps 3–9 are being repeated *I* times.

**Figure 1:**
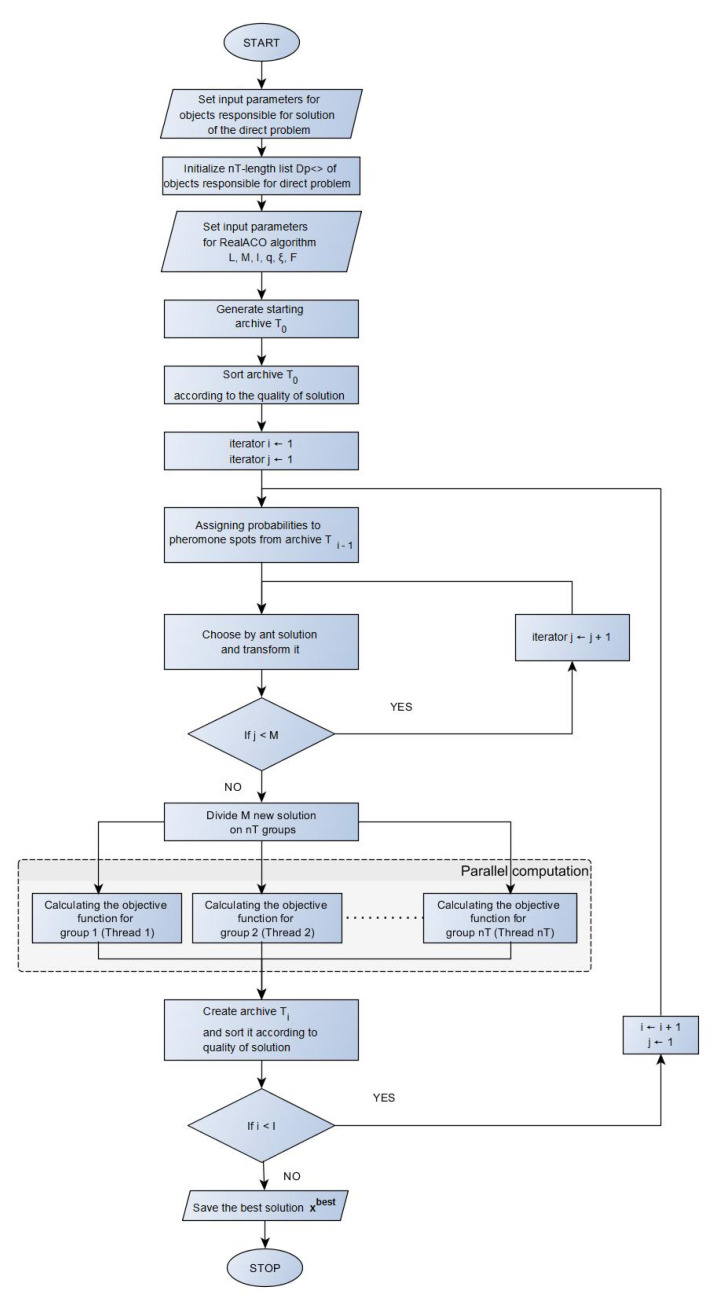
The block diagram of the Real Ant Colony Algorithm.

Knowing the values of the parameters L,M and *I* we can determine the number of the objective function calls during the algorithm execution. This number is L+M·I. The Figure 1 presents the block diagram of the RealACO algorithm.

## 4. Numerical Examples

In this section, we present two numerical examples that we used for comparing the algorithm proposed by the authors (based on the Real Ant Colony Optimization algorithm) with the iteration algorithm presented in the paper [18]. In both examples we are considering the model (1)–(4), and the reconstructed coefficient is thermal conductivity λ dependent on time. The input data for the inverse problem is a set of values $b^{k}$ (6).

### 4.1. Example 1

For this example we take the following data:

f(x)=0,ϕ(t)=ψ(t)=0,α=0.9,c=ϱ=1,

$$L=T=1,\;\;a\left(t\right)=t^{2}+t,\;\;g\left(x,t\right)=0.105114\left(10t^{0.1}+18.1818t^{1.1}\right)\left(x-1\right).$$

The reconstructed coefficient λ is in the following form:

$$\lambda \left(t\right)=\left\{\begin{array}{cc} a_{1} & \;\;\;t\in (0,\frac{1}{3}),\\ a_{2} & \;\;\;t\in [\frac{1}{3},\frac{2}{3}),\\ a_{3} & \;\;\;t\in [\frac{2}{3},1]. \end{array}\right.$$

The exact values of the parameters $a_{1}$, $a_{2}$, $a_{3}$ are 1, 0.5, 1. For minimizing the functional (7) the RealACO was applied with the parameters L=6, M=15, I=40. The values of the parameters were chosen basing on the experiments that we had carried out earlier. The number of the objective function calls is 606. Table 1 presents the results of the reconstruction.

The error of the obtained results was calculated according to formula

$$\mathrm{error}=\sqrt{\sum_{k=1}^{N}{\left|{\bar{\lambda }}^{k}-\lambda^{k}\right|}^{2}},$$

where *N* indicates the number of measurements, $\bar{\lambda }$ is the reconstructed coefficient and λ is the exact value of the coefficient. The grid used to generate the input data was of the size 100×900 as well as the grid used in the algorithm for solving the direct problem. Table 2 presents the results in dependence on the size of input data disturbance.

In case of the exact input data the smaller value of the parameter ϵ is, the smaller error of the result obtained by the iteration algorithm we get. For ϵ=0.001, the approximate error is $4.1\times 10^{-4}$, while for ϵ=0.0001, the error is $3.56\times 10^{-5}$. In case of the RealACO algorithm the obtained error is approximately $4.28\times 10^{-4}$ and it is slightly bigger than the error obtained by the iteration algorithm. For the input data disturbed by the pseudorandom error decreasing the parameter ϵ in the iteration method slightly reduces the errors of the obtained results. In case of the disturbed input data the reconstruction errors of the thermal conductivity coefficient λ when using the algorithm proposed in the paper were smaller than when using the iteration method. For the data disturbed by the 5% pseudo-random error the RealACO algorithm reconstructed the λ coefficient with the error $8.7965\times 10^{-3}$ while for the iteration method the error was $4.4012\times 10^{-1}$.

### 4.2. Example 2

The input data used in the previous example were taken from the paper [18]. On the contrary, this example was not presented in that paper. In this example we compare both algorithms with the input data generated on a grid of a different density than the grid used in the algorithms reconstructing the λ coefficient. In such a case, testing the algorithm is a base for avoiding the inverse crimes [33] (page 5). The grid used for generating the input data was of size 100×2000 and the grid used in the reconstruction process was of size 100×1800. The algorithm described in the paper [18] was implemented by us in the C# and then the computation was carried out. The results are presented in the Table 3.

The computational data are as follows.

f(x)=0,ϕ(t)=ψ(t)=0,α=0.9,c=ϱ=1,

$$L=T=1,\;\;a\left(t\right)=t^{2}+t,\;\;g\left(x,t\right)=0.105114\left(10t^{0.1}+18.1818t^{1.1}\right)\left(x-1\right).$$

The λ coefficient is being reconstructed in the following form,

$$\lambda \left(t\right)=a_{1}\sin\left(a_{2}\pi t\right)+a_{3},$$

where the exact values of the parameters $a_{1}$, $a_{2}$, and $a_{3}$ are 1, 4, and 1.1, respectively. In the RealACO algorithm, the parameters M=16, L=12, and I=35 were used. The values of the parameters were chosen basing on the experiments that we had carried out earlier. Table 4 presents the results obtained for the RealACO algorithm.

The result of the reconstruction obtained from the RealACO algorithm are very well, the most reconstruction errors are smaller than the input data errors. Even for the input data disturbed by the 5% error the reconstruction errors are no more that 1.19%. Moreover, the values of the standard deviation for the obtained results are low.

Table 3 presents the results used for comparison of the algorithms for various disturbances of the input data. In case of the iteration method the change of the ϵ parameter insignificantly affects the reconstruction errors of the coefficient λ. In each considered case the reconstruction errors of the λ coefficient are smaller for the RealACO algorithm than for the iteration method.

**Figure 2:**
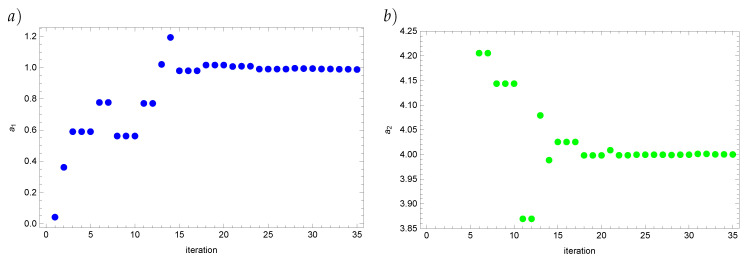
Values of the coefficients ${\bar{a}}_{1}$ (**a**) and ${\bar{a}}_{2}$ (**b**) for the input data disturbed by the 5% error.

**Figure 3:**
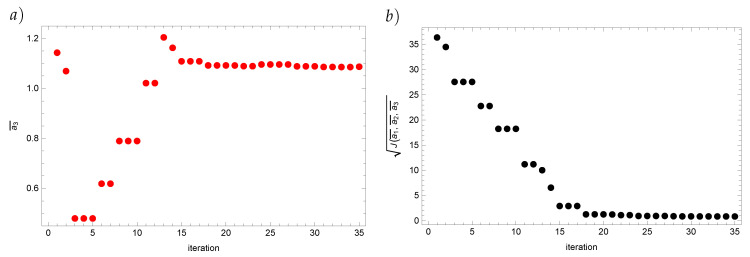
Value of the coefficient ${\bar{a}}_{3}$ (**a**) and value of the functional *J* (**b**) for the input data disturbed by the 5% error.

Finally, we present how the solutions in the respective iterations converge to the approximate solution for the Real Ant Colony Optimization algorithm (Figure 2 and Figure 3). From about the twentieth iteration the value of the reconstructed coefficient as well as the value of the functional start changing insignificantly.

## 5. Conclusions

The algorithm for solving the inverse problems on the models described by the differential equation with the Caputo type derivative presented in the paper seems to be an effective tool for solving these type of problems. The presented method comes to minimizing the functional representing the approximation error. For the functional, the classical methods of finding the extrema cannot be used and because of that the Real Ant Colony Optimization algorithm was used in the paper. The reconstruction errors obtained in the presented examples were small and did not exceed the input data disturbances. Additionally, the presented method turned out to be more precise than the method presented in the article [18], especially assuming that the input data was generated on different grid than the grid used for solving the direct problem.

## Figures and Tables

**Table 1:** Results of calculations for the RealACO algorithm (grid 100×900) (${\bar{a}}_{i}$—reconstructed value of the coefficient $a_{i}$, $\delta_{{\bar{a}}_{i}}$—the relative error of reconstruction of the coefficient $a_{i}$, σ—standard deviation (i=1,2,3)).

Noise	${\bar{a}}_{i}$	$\delta_{{\bar{a}}_{i}}\left[\%\right]$	σ
0%	0.9999	0.01	$4.36\times 10^{-4}$
	0.4999	0.02	$7.81\times 10^{-5}$
	0.9999	0.01	$4.36\times 10^{-5}$
1%	1.0001	0.01	$1.77\times 10^{-4}$
	0.4998	0.04	$1.74\times 10^{-4}$
	0.9999	0.01	$7.21\times 10^{-5}$
2%	0.9995	0.05	$1.96\times 10^{-4}$
	0.4996	0.08	$3.67\times 10^{-4}$
	0.9997	0.03	$1.96\times 10^{-5}$
5%	1.0001	0.01	$3.79\times 10^{-4}$
	0.4996	0.08	$5.82\times 10^{-5}$
	1.0003	0.03	$4.84\times 10^{-5}$

**Table 2:** Comparison of the algorithms according to the obtained errors of reconstructing the coefficient λ for the input data from the example 1.

Noise	RealACO	Iteration Method	
	Error	ε	Error
0%	$4.2781\times 10^{-4}$	0.01	$4.8035\times 10^{-3}$
		0.001	$4.0918\times 10^{-4}$
		0.0001	$3.5545\times 10^{-5}$
1%	$3.3106\times 10^{-3}$	0.01	$9.0230\times 10^{-2}$
		0.001	$8.9619\times 10^{-2}$
		0.0001	$8.9578\times 10^{-2}$
2%	$1.0889\times 10^{-2}$	0.01	$1.8033\times 10^{-1}$
		0.001	$1.8057\times 10^{-1}$
		0.0001	$1.8059\times 10^{-1}$
5%	$8.7965\times 10^{-3}$	0.01	$4.4052\times 10^{-1}$
		0.001	$4.4016\times 10^{-1}$
		0.0001	$4.4012\times 10^{-1}$

**Table 3:** Comparison of the algorithms according to the obtained errors of reconstructing the coefficient λ for the input data from example 2.

Noise	RealACO	Iteration Method	
	Error	ε	Error
0%	0.6230	0.01	1.1821
		0.001	1.1825
		0.0001	1.1826
1%	0.5982	0.01	1.2050
		0.001	1.2054
		0.0001	1.2055
2%	0.6146	0.01	1.2369
		0.001	1.2374
		0.0001	1.2375
5%	0.6566	0.01	1.4903
		0.001	1.4907
		0.0001	1.4908

**Table 4:** Results obtained by the RealACO algorithm (grid 100×1800) (${\bar{a}}_{i}$ – reconstructed value of a coefficient $a_{i}$, $\delta_{{\bar{a}}_{i}}$ – relative error of reconstruction of the coefficient $a_{i}$, σ – standard deviation (i=1,2,3)).

Noise	${\bar{a}}_{i}$	$\delta_{{\bar{a}}_{i}}\left[\%\right]$	σ
0%	0.9889	1.10	0.1077
	3.9999	0.01	0.0124
	1.0875	1.14	0.0507
1%	0.9910	0.90	0.0128
	3.9991	0.03	0.0040
	1.0874	1.15	0.0140
2%	0.9900	0.99	0.0266
	3.9998	0.01	0.0076
	1.0873	1.15	0.0156
5%	0.9881	1.18	0.0277
	4.0002	0.01	0.0182
	1.0869	1.18	0.0150
